# High doses of CRISPR/Cas9 ribonucleoprotein efficiently induce gene knockout with low mosaicism in the hydrozoan *Clytia hemisphaerica* through microhomology-mediated deletion

**DOI:** 10.1038/s41598-018-30188-0

**Published:** 2018-08-06

**Authors:** Tsuyoshi Momose, Anne De Cian, Kogiku Shiba, Kazuo Inaba, Carine Giovannangeli, Jean-Paul Concordet

**Affiliations:** 1 Sorbonne Université, CNRS, Laboratoire de Biologie du Développement de Villefranche-sur-Mer (LBDV) 181 Chemin du Lazaret, 06230 Villefranche-sur-Mer, France; 2 Laboratoire Structure et Instabilité des Génomes, INSERM U1154, CNRS UMR7196, Museum National d’Histoire Naturelle 43 rue Cuvier, 75005 Paris, France; 3 Shimoda Marine Research Centre, University of Tsukuba, 5-10-1 Shimoda, Shizuoka, 415-0025 Japan

## Abstract

Targeted mutagenesis using CRISPR/Cas9 technology has been shown to be a powerful approach to examine gene function in diverse metazoan species. One common drawback is that mixed genotypes, and thus variable phenotypes, arise in the F0 generation because incorrect DNA repair produces different mutations amongst cells of the developing embryo. We report here an effective method for gene knockout (KO) in the hydrozoan *Clytia hemisphaerica*, by injection into the egg of Cas9/sgRNA ribonucleoprotein complex (RNP). Expected phenotypes were observed in the F0 generation when targeting endogenous GFP genes, which abolished fluorescence in embryos, or *CheRfx1*23 (that codes for a conserved master transcriptional regulator for ciliogenesis) which caused sperm motility defects. When high concentrations of Cas9 RNP were used, the mutations in target genes at F0 polyp or jellyfish stages were not random but consisted predominantly of one or two specific deletions between pairs of short microhomologies flanking the cleavage site. Such microhomology-mediated (MM) deletion is most likely caused by microhomology-mediated end-joining (MMEJ), which may be favoured in early stage embryos. This finding makes it very easy to isolate uniform, largely non-mosaic mutants with predictable genotypes in the F0 generation in *Clytia*, allowing rapid and reliable phenotype assessment.

## Introduction

Gene editing is now possible in a wide range of organisms by exploiting the molecular components of the CRISPR bacterial adaptive immune system^1–5^. The well-known SpCas9 protein from *Streptococcus pyogenes* acts as a sequence specific exonuclease^6,7^. Cas9 protein in a complex with a short guide RNA (sgRNA) stimulates RNA-DNA duplex formation between the sgRNA and a target sequence followed by a PAM motif (NGG for SpCas9), and then makes a double strand break (DSB) 3 or 4 bp upstream of the PAM motif^ 8^. Gene editing *per se* is achieved during repair of the DSB caused by the Cas9/sgRNA complex. Small insertions or deletions can be introduced as a result of errors during DNA repair, while precise modifications can be obtained by homology-directed DNA repair with an exogenous DNA template^9–12^. Gene editing is thus strongly dependent on the activity of endogenous DSB repair machinery in the targeted cells.

Gene knockout (KO) induced by CRISPR/Cas9 has now been demonstrated in a wide range of metazoan species, including many for which classical genetic approaches are not feasible^13–29^. This is allowing researchers to address a wide range of questions, for instance in the fields of ecology, evolution and biodiversity, that would be difficult to tackle in the common genetics model organisms such as *Drosophila* or zebrafish.

A common approach for CRISPR/Cas9-mediated gene KO is to introduce the Cas9/sgRNA RNP complex, or DNA/RNA encoding its components, into the egg cytoplasm by microinjection. The resultant embryos usually comprise a “mosaic” of different genotypes resulting from independent mutations in different cell linages. A pitfall of this approach is thus that phenotypes vary between embryos depending on the mix of induced genotypes, even when the overall mutation efficiency is high, for instance if some cell populations have in-frame mutations with little or no functional consequence for the protein produced. In mammalian embryos, the frame-shift mutations can be favoured by computationally choosing sgRNAs based on the presence of sequence microhomologies flanking to the DSB site^30,31^. This strategy relies on the prevalence of microhomology-mediated end-joining (MMEJ) repair mechanism in mammalian embryos, which promotes frequent deletions between these sites^17,21,30^. In *Xenopus* another strategy used to create non-mosaic F0 heterozygotes is to inject Cas9/sgRNA into oocytes before meiotic maturation and fertilization, thus favouring mutation before cleave divisions begin^32^.

**Figure 1:**
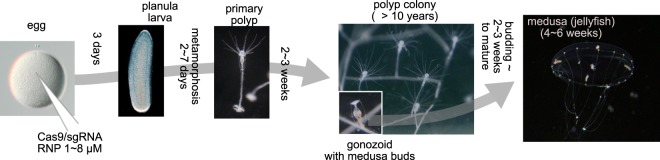
Life cycle of *Clytia hemisphaerica*. Cas9 RNP is injected into unfertilized eggs, which develop to planula larvae in 3 days after fertilization. Planulae undergo metamorphosis to make polyp colonies, which propagate vegetatively and thus constitute a genetic resource convenient to maintain and replicate. The colony releases juvenile jellyfish, which will grow to sexual maturity in a few weeks and spawn every day. The minimum life cycle is 2~3 months.

Here we report a highly efficient gene KO method for the hydrozoan jellyfish *Clytia hemisphaerica*, which exploits particularities both of the DSB repair pathway deployment during embryogenesis, and its characteristic hydrozoan life cycle. In *Clytia* the fertilized egg develops in three days into a planula larva, which then metamorphoses to generate a quasi-immortal, vegetatively propagating colony of connected polyps. Sexually reproducing jellyfish bud continuously from specialised polyps of the colony (Fig. 1)^33,34^. We achieved highly efficient biallelic mutation for two endogenous Green Fluorescent Protein (GFP) genes by Cas9/sgRNA RNP injection into the egg. Similar success was achieved for the *CheRfx123* gene, the single *Clytia* counterpart of vertebrate *Rfx1*, *2* and *3* genes that play conserved roles for cilia formation in bilaterians and observed sperm flagella defects in jellyfish generated from F0 polyps. Cas9/sgRNA injected embryos and larvae were mosaic, but we uncovered an unexpected enrichment phenomenon during the life cycle such that mutant jellyfish had very low mosaicism with predominant deletions between microhomologies. By exploiting this unique phenomenon, it is possible to make non-mosaic *Clytia* mutant polyps and jellyfishes in the F0 generation.

## Results

### Highly efficient biallelic gene KO by CRISPR/Cas9 in *Clytia*

**Figure 2:**
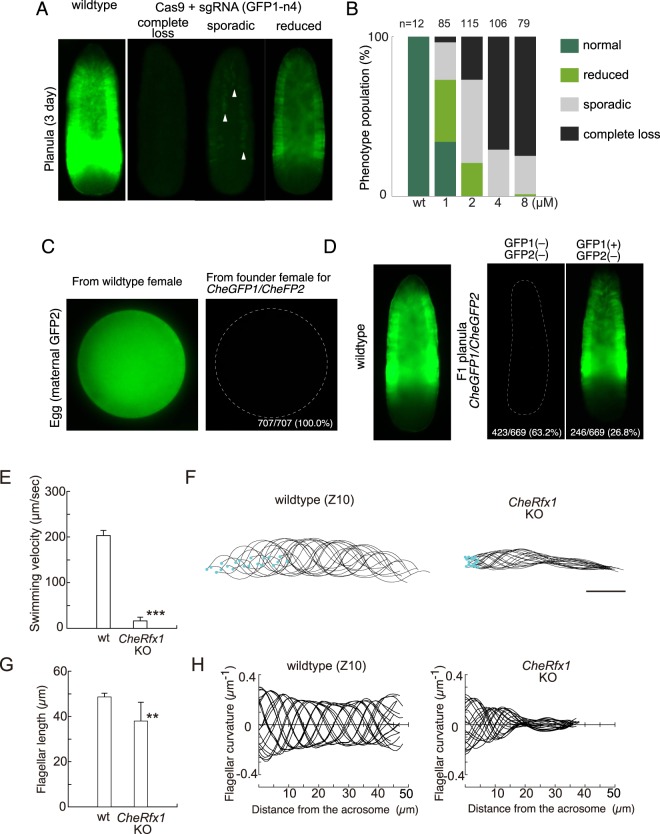
Highly efficient gene KO by CRISPR/Cas9 in *Clytia hemisphaerica*. (**A**) Classification of endogenous green fluorescence depletion phenotypes in Cas9/sgRNA(GFP1n4) RNP-injected 3-day old planula larvae. “wildtype” shows typical endogenous green fluorescence, mainly from *CheGFP1* gene. In “complete loss” no green signal was observed except for uniform maternal GFP signal. In “sporadic”, groups of weakly GFP positive cell form patches (arrowhead), each probably descended from single cells in which *CheGFP1* was mutated only after the onset of transcription. An embryo was classified as having “reduced” green fluorescence if the signal was significantly reduced but still showed the typical regional expression pattern. (**B**) Population of phenotypes described in (**A**) for each Cas9 RNP concentration tested. (**C**) Maternal CheGFP2 fluorescence in eggs released from wildtype and *CheGFP1/ CheGFP2* double KO F0 jellyfish. (**D**) GFP fluorescence in a wildtype planula (left) and in F1 planulae from male and female *CheGFP1*/*CheGFP2* double KO founder jellyfish, which were either completely GFP free (*CheGFP1*^−/−^ centre) or almost identical to wildtype (*CheGFP1*^+/−^ or *CheGFP1*^+/+^, right). In both cases, functional maternal CheGFP2 protein was absent according to (**C**). (E~G) Sperm motility defect in sperm produced *CheRfx123* KO F0 jellyfish observed by speed microscopic movies for wildtype (N = 6) and *CheRfx123*-KO (N = 8) sperms. (**E**) Swimming velocity (***p < 0.001 Welsh’s t-test). (**F**) An example of sperm flagellar bending forms by time. 20 bending forms of a single sperm were picked out at every 5 milliseconds (0.1 seconds in total) and superimposed. Blue dots show the position of sperm head. *CheRfx1* KO sperm oscillated but barely translocated. (**G**) Flagellar length (**p < 0.01 Welsh’s t-test). (**H**) An example of bending curvature of sperm flagella, superimposed for 20 bending forms with 5 milliseconds of interval. The x-axis is the distance along the flagellum from its base and y-axis is for the curvature at the point indicated by µm^−1^, which is reciprocal of the radius of the circle that fits a curve. In *CheRfx1* KO sperm, the wave of sperm bending did not propagate long distance from the base of flagellum.

To test CRISPR/Cas9 knockout protocols in *Clytia* we performed pilot studies on genes predicted to have easily assessible phenotypes, the endogenous GFP genes and a *Clytia* orthologue of the RFX family of transcription factors, *CheRfx123*. Like the hydrozoan jellyfish *Aqueora victoria*^35^ from which GFP was originally isolated, *Clytia* larvae and medusa produce green fluorescent proteins from endogenous GFP genes, providing convenient targets for the development of gene KO approaches. Four *Clytia* GFP genes (*CheGFP1*-*CheGFP4*) with distinct expression profiles have been reported^36^. In planula larvae, *CheGFP1* expressed from the zygotic genome produces a band of green fluorescence in the lateral ectoderm, while maternal CheGFP2 protein, targeted to egg mitochondria, accounts for weaker, uniform expression. *CheGFP3* and *CheGFP4*, in contrast, are expressed predominantly in adult cell types, with little or no mRNA detectable at embryonic and larval stages. We thus selected *CheGFP1* as an ideal target to test gene KO in *Clytia*. NLS-tagged SpCas9 (Cas9) protein and single guide RNA (sgRNA) were pre-assembled and microinjected into eggs prior to fertilisation. Out of three sgRNAs tested, GFP1n4 provoked a loss of GFP at the planula stage (Fig. 2A). CheGFP1 fluorescence was completely or almost completely abolished in the planula larvae 3 days after fertilisation when 4 µM or higher Cas9 RNP concentration was microinjected (Fig. 2B). Remaining uniform and faint green fluorescence in CheGFP1 “complete loss” embryo (Fig. 2A) probably reflects the presence of residual maternal CheGFP2. Some specimens in the high Cas9 RNP condition showed sporadic and weak CheGPF1 signal, which was often in longitudinal stripes of cells (arrowhead in “sporadic” Fig. 2A), suggesting each line was clonally derived from a single blastomere at the blastula stage. Another sgRNA, GFP1n1, did not alter CheGFP1 fluorescence in planula, even though it induced mutations at high rates (9/13 at 3 µM). To generate *Clytia* strains lacking GFP expression during embryonic development as a resource for future studies using exogenous EGFP or EYFP reporters, we targeted the two maternal/embryonic GFP genes *CheGFP1* and *CheGFP2* simultaneously by injecting a mixture of two sgRNAs (GFP1n4 and GFP2n7) and Cas9. 3 days after injection the planulae were induced to metamorphose into primary polyps, which were then reared to give F0 polyp colonies generating jellyfish 1–2 months after injection. When high concentrations of Cas9 RNP were used, none of the eggs collected from F0 jellyfish showed CheGFP2 expression (Fig. 2C). 63.2% (n = 669) of F1 larvae obtained by crossing male and female F0 founder jellyfish lacked CheGFP1/CheGFP2 fluorescence (Fig. 2D). This result shows highly efficient mutation in the jellyfish germline. We were able to establish 6 GFP mutant colonies (2 males and 4 females, *CheGFP1*^−/−^; *CheGFP2*^−/−^). No visible phenotype was detected during embryogenesis in the GFP-free F1 generation, except for the loss of the green fluorescence.

Another pilot target gene was *CheRfx123*, a *Clytia* orthologue of the Rfx (Regulatory Factor X) family of genes that encode transcription factors playing evolutionary conserved roles for ciliogenesis in bilaterians^37^. Vertebrates generally have three paralogous Rfx genes *Rfx1, 2 and* 3. *Rfx2* and *Rfx3* mutants showed various defects in cilia (dysfunction of motile cilia, fewer or truncated motile cilia,^38–40^ and in the nematode *C. elegans* mutants of the Rfx gene *DAF-19* lack all sensory neuron cilia^41^. We reasoned that *CheRfx123* mutants would be useful for addressing the role of cilia in embryonic and larval development, during which the alignment of monociliated ectoderm cells becomes coordinated along the developing body axis^42^, and might also provide a useful resource to generate non-motile embryos for microscopy. Animals injected with Cas9/sgRNA(Rfxn1) RNP showed no visible phenotype of the motile cilia in the planula ectoderm or in the jellyfish gonad ectoderm (data not shown), despite high efficiency of biallelic mutation (see following section). Strikingly, however, F0 male jellyfish were completely sterile and showed a clear defect in the sperm motility (Fig. 2E,F). Sperm locomotion was significantly slower. Movie image analysis showed that flagella bending did not propagate more than 20 µm from the base of the flagella (Fig. 2H). The length of the sperm flagella also seemed significantly reduced compared to wild type (Fig. 2G). No fertilisation was achieved with the sperm from *CheRfx123*-KO F0 jellyfish, indicating complete absence of any functional spermatozoids.

### Most Cas9 induced deletions in *Clytia* polyps and jellyfish are between microhomologies

**Figure 3:**
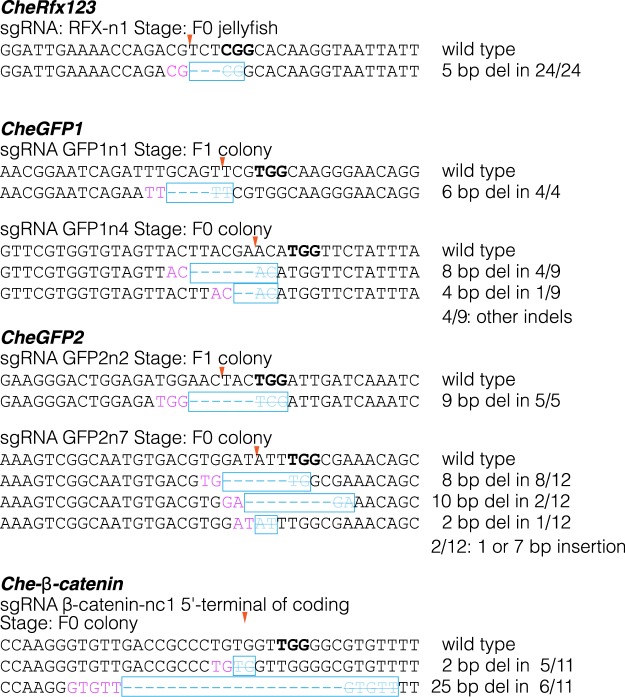
Prevalence of MM deletion in polyp and jellyfish stages. Mutation and their frequencies observed by Sanger sequencing of PCR clones from polyp and jellyfish stages are indicated. Complete or highly prevalent MM deletions were observed in all 6 targets from 4 genes. sgRNA sequence is underlined and PAM motif is represented in bold. Left microhomology sequences are indicated by magenta characters. The magenta arrow and cyan box indicate respectively, the predicted double-strand break position and deleted sequence in the mutant. Stage and frequency are indicated on the right.

**Figure 4:**
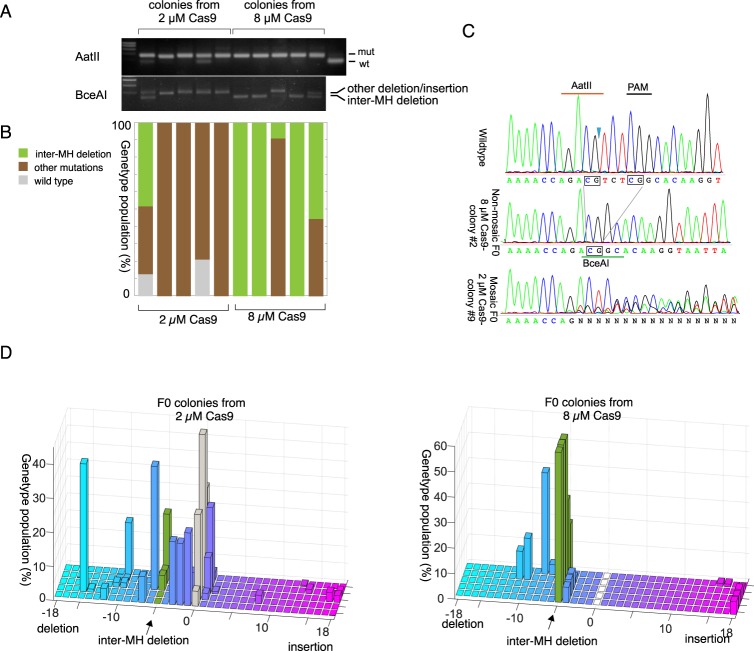
Creation of non-mosaic *CheRfx123* KO colonies in F0 generation at high dose of Cas9 RNP. (**A**,**B**) Estimation of the genotype population by digestion of PCR products with restriction enzymes, AatII (top gel) and BceAI (bottom gel), that cut wildtype sequence and the 5 bp MM deletion respectively. (**A**) Agarose gel electrophoresis of digested PCR products. AatII-resistant bands showed nearly complete biallelic mutation at both concentrations. Complete BceAI digestion of PCR product from three out of five colonies obtained after injection of 8 µM Cas9 showed that they uniformly consisted in the 5 bp MM deletion. The analysis was repeated for 5 independent colonies for each condition. (**C**) An example of sequence chromatogram for direct sequencing of PCR product, showing uniform wildtype colony (top), uniform MM deletion colony (middle, at high Cas9 RNP concentration) and a mosaic mutant colony (bottom, at low Cas9 RNP concentration). The DSB site is indicated by a triangle. The microhomology sequences are surrounded by boxes. The PAM motif and AatII/BceAI site used in restriction enzyme analysis are indicated by bars. (**D**) Estimation of frequency of indel by sizes (lengths) by TIDE calculations. The estimation in the 5 different polyp colonies (y-axis) for low (2 µM) and high (8 µM)Cas9 RNP conditions. Deletions and insertions are to the left and right of the wild type (0 bp indels, grey) in x-axis. The 5 bp deletion corresponding MM deletion is marked by arrows (green columns).

From genotype analysis at the F0 polyp or jellyfish stages or in the F1 generation, we noticed that one or two specific deletions always dominated (Fig. 3A). For example, 13 out of 20 randomly sequenced CheRfx123 PCR clones from four polyp colonies (5 clones/colony) had an identical 5 bp deletion, and otherwise had an insertion in addition to the 5 bp deletion (6/20). All 24 clones from six jellyfishes (4 clones/jellyfish) had the same 5 bp deletion. A similar low diversity of mutations in F0 polyps and in the F1 generation was observed with all target sgRNAs tested for *CheGFP1* and *CheGFP2* genes using the same injection conditions (Fig. 3B). Similarly, mutations induced at the 3′-end of *Che-β-catenin* coding region consisted only of either 5 or 25 bp deletions when examined in F0 polyp colony (Fig. 3B). Strikingly, sequence analysis revealed that for all genes tested these dominant deletions had taken place between two identical 2 to 5 bp sequences, so called microhomologies, lying on either side of the double strand break (DSB) (Fig. 3). We will refer to this type of deletion as microhomology mediated (MM) deletions in this work. Previous studies suggested that MM deletions are caused by DSB repair by the MMEJ pathway^43,44^. To investigate the basis of the prevalence of the MM deletions, we focused our analyses on the *CheRfx123* gene, in which the dominant 5 bp deletion can be detected by restriction enzyme digestion. To test Cas9/sgRNA-dose dependence, we injected low (2 µM) and high (8 µM) concentrations of Cas9 RNP into eggs and established several polyp colonies for each condition. After 4 months of culture, genomic DNA was extracted from 5 randomly selected polyp colonies and the genotypes within each colony were examined. Digestion of PCR products amplified from the polyp colony by restriction enzymes AatII and BceAI, which selectively cut wildtype and the 5 bp MM deletion respectively, showed that 3 out of 5 F0 colonies from the high Cas9 RNP condition (8 µM) were uniformly comprised of cells with the 5 bp MM deletion (Figs 4A,B and S2). No uncut PCR product was detectable by BceAI, indicating that virtually 100% of *CheRfx123* copies in these colonies had MM deletion. The other two colonies were mosaic, showing 9% and 56% copies of the MM deletion. At lower concentration of Cas9 RNP, KO efficiency was still high. Three colonies still showed 100% KO efficiency and the remaining two had at least 79% efficiency. However, in contrast to the injections at high Cas9 RNP concentration, no MM deletion could be detected in low Cas9 RNP condition in 4 out of 5 colonies, with the fifth colony showing 49% MM deletion.

Uniform genotypes in the three colonies generated from high-dose Cas9 injections were visible in the chromatogram of direct sequencing of the PCR products (Fig. 4C). TIDE (Tracking of Indels by DEcomposition) analysis, which breaks down the mixed indel compositions from sequence trace data^45^, confirmed that polyp colonies derived from high concentration Cas9 RNP injections were dominated by the 5 bp MM deletion (Fig. 4D). In contrast, mutations were more diverse in polyp colonies derived from low concentration Cas9 RNP injection, and the MM deletion was not predominant (Fig. 4D). These analyses demonstrate that non-mosaic and homozygous *Clytia* mutant polyp colonies can be made in a single generation simply by injecting high concentrations of Cas9/sgRNA RNP, and that MM deletions are favoured in these conditions.

### MM deletions are prevalent at the planula stage and become enriched in polyps

**Figure 5:**
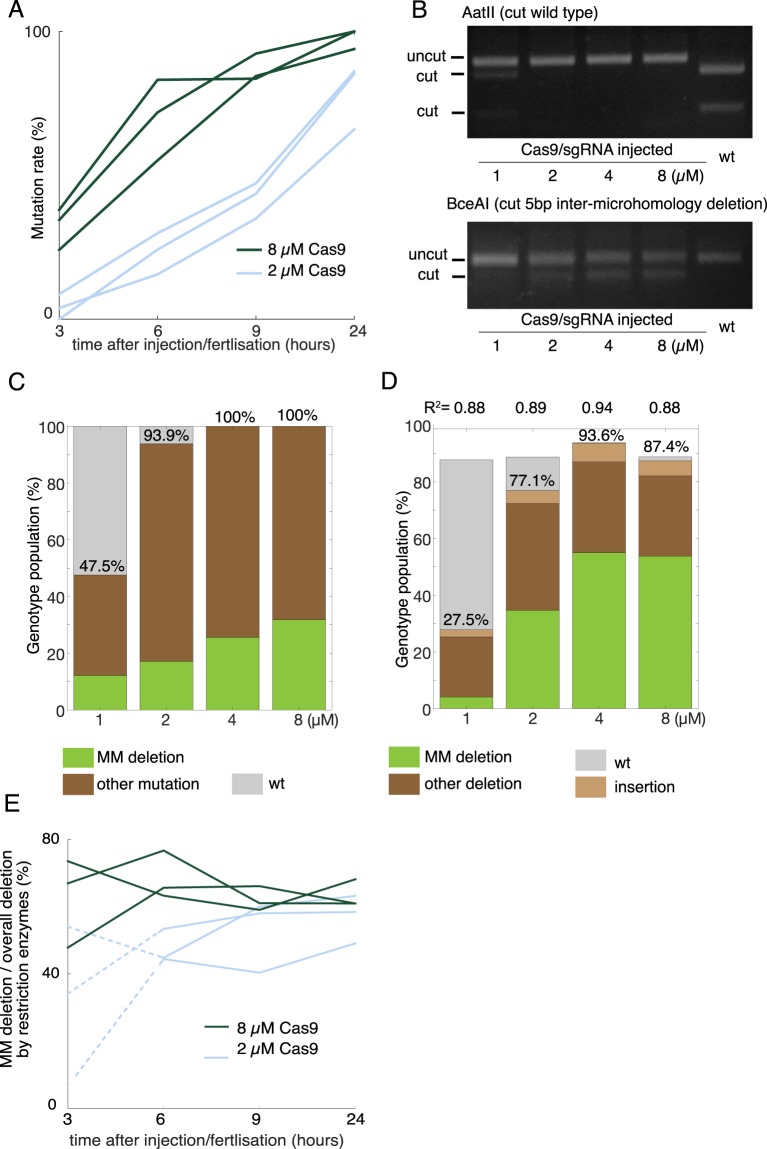
Target sequence mutation rate and MM deletion frequency after injection with different doses of Cas9/sgRNA RNP. (**A**) The time course of *CheRfx123* KO efficiency after Cas9/sgRNA injection (2 µM and 8 µM). KO efficacy was measured by AatII digestion from three independent experiments for each condition. (**B**) Agarose gel electrophoresis of AatII/BceAI digested PCR products to measure KO efficiency and the rate of MM deletion in 3-day planula larvae. (**C**,**D**) Population of genotypes in Cas9-injected 3-day planulae estimated by restriction enzyme digestion (**C**) and by TIDE (**D**) of the PCR products. Average of three independent injections. Concentration dependency of MM deletion frequency over all detected mutation was confirmed by 1-way ANOVA with p = 0.00116 for (**C**) and p = 2.75e-05 for (**D**). wt: grey, MM deletion green other brown. Numbers on the bar indicate average mutation rate (%). Numbers on above (**D**) are coefficient of determination (R^2^) of non-linear least square analysis used for TIDE calculation, indicating how well the estimated fits to the observed chromatograms. In practice, it matches to the sum of mutated and unmutated DNA sequences. (**E**) Time-course observation of MM deletion frequency from 3 to 24 hours after injection with low concentration (2 µM light blue) and high concentration (8 µM dark green) of Cas9 RNP in three independent experiments. The measurement was not reliable at 3 hpf with lower Cas9 RNP concentration due to very low mutation rate, which are thus indicated by the dotted line.

To address carefully how and when MM deletions took place, we tested the timing of mutagenesis after Cas9 RNP injection by digesting target PCR products by AatII. Following injection of Cas9 RNP at high concentrations (8 µM), a large proportion of the target copies (average 87%) were modified as early as 9 hours after injection, and virtually all target copies (98%) were modified within 24 hours (Fig. 5A). In comparison indel rates induced by the lower Cas9 concentration (2 µM) were on average 42% at 9 hours and 80% at 24 hours. The timing of mutagenesis was thus Cas9 RNP dose-dependent. In parallel, we examined by restriction enzyme digestion and TIDE analysis the frequency of overall mutations and MM deletions induced by different Cas9 RNP concentrations at the 3-days-old planula stage, when they become ready to undergo metamorphosis into polyps (Fig. 5B–D, and S2). As assessed by AatII digestion, 93% mutants using 2 µM Cas9 RNP and no wildtype CheRfx123 copies was detectable when 4 µM or higher Cas9 RNP concentration was used (Fig. 5B,C). TIDE analysis similarly detected few or no wildtype *CheRfx123* copies following injection of Cas9 RNP at 4 µM or higher (Fig. 5D). This assay generally estimated higher wildtype frequencies than restriction enzymes, for example 12% by TIDE versus 6% by AatII digestion in larvae generated by injection of 2 µM Cas9 RNP). The estimated frequency of MM deletion was also different between TIDE analysis and digestion with restriction enzymes (58% vs 33% respectively using 8 µM Cas9 RNP). The rate of MM deletion relative to overall mutations was, however, clearly Cas9-dose dependent with both analyses (Figs 5C,D and S1). To explain this clear trend, we hypothesised that MM deletions might be favoured at the earlier times during development. Comparison of MM deletion frequency between the earlier and later stages did not, however, reveal any temporal shift during development (Fig. 5E).

**Figure 6:**
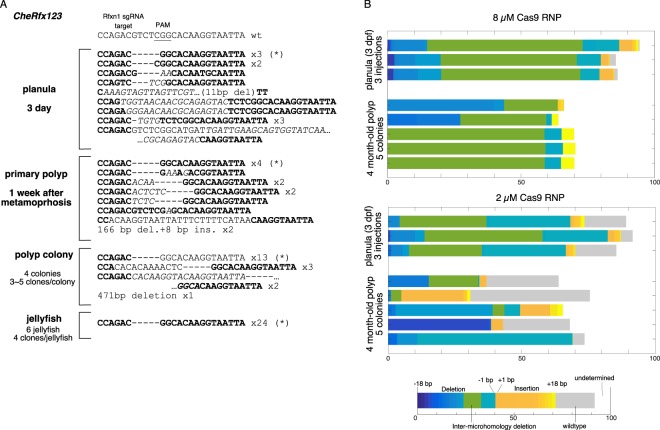
Increasing prevalence of MM deletion from planula to polyp/jellyfish stage in *CheRfx123* gene. Mutations observed by Sanger sequencing of PCR clones from different stages. Bold and italic represent nucleotide residues conserved with wildtype and insertion respectively. (**A**) deletion is indicated with a hyphen. Numbers indicate the frequency of identical genotype in each sequencing set. The dataset is from the same series of the injection as Figs 2 and 3. (**B**) TIDE breakdown of mutant genotype population analysed in 3-dayold planula larvae from separate 3 injections and 5 individual colonies of polyp 4-months old colonies after injection of high (top) and low (bottom) Cas9-RNP concentration. The dataset is from the same series of injections as Figs 4 and 5.

Finally, to understand the relationship between the MM frequency observed at planula stages and later polyp colonies, we compared genotype shift between these stages for the same set of mutagenesis experiments (Fig. 6). Sanger sequencing confirmed that MM deletion is already slightly more common than other mutations in the mosaic planula larvae, and then these then become highly dominant in F0 planula and jellyfish stages (Fig. 6A). MM deletion was gradually enriched as the life cycle progressed. Systematic analysis using TIDE following high concentration Cas9 RNP injections confirmed this result (Fig. 6B). In contrast, following low concentration Cas9 RNP injections, MM deletions were slightly less frequent in planula larvae and then disappeared from the colony. This result suggests that polyp-forming stem cells, which are only a small part of planula larvae, have different mutation profiles as compared to somatic cells. The stem cell may therefore be highly sensitive to Cas9 RNP dose to cause the MM deletion.

## Discussion

We demonstrated that gene KO using CRISPR-Cas9 is highly efficient and effective in the hydrozoan *Clytia hemisphaerica*, as previously reported in many other metazoans. Importantly, it is feasible in *Clytia* to generate a nearly non-mosaic mutant polyp colony in the F0 generation. When microinjecting high concentrations of Cas9 RNP, one or two deletions that occur specifically between pairs of sequence microhomologies become enriched in the polyp colony. The advantages of this low-mosaicism in F0 animals was well illustrated by the CheRfx123-KO, which showed a sperm motility defect and thus was sterile. It is therefore feasible in *Clytia* to rapidly test the role of genes that are critical for gametogenesis and early embryonic development. The relatively short life cycle (2~3 months) of *Clytia* also allows a more standard genetic approach as demonstrated here by generating GFP1/GFP2 double KO in F1. The vegetative growth of the polyp colony makes *Clytia* a unique genetic model animal. The mutant colonies created by our approach, including weakly-mosaic F0 colonies, can in principle, like wild-type colonies, be maintained for at least five to ten years simply by daily feeding with *Artemia salina* larvae. This approach is particularly useful for functional studies of genes involved in reproduction^46^. The mutant colonies can be replicated by transplanting polyps to new colony plates. *Clytia* mutant strains are therefore very easy to share in the research community.

**Figure 7:**
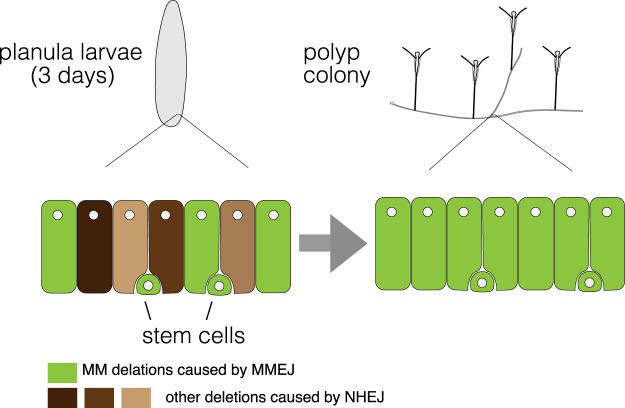
A scenario explaining how MM deletion could become dominant in polyp colonies generated from mosaic planula larvae. As polyp colonies grow in vegetative manner, massive proliferation of founder cells occurs. If MMEJ pathway is favoured in putative founder stem cells, resulting in the creation of MM deletions, these will become dominant in the polyp colony, from an initially mosaic planula larvae.

In most animal species, the type of mutation induced by Cas9 RNP is variable, and highly mosaic in the mutant F0 generation^44^. In *Clytia*, following injection of low doses of Cas9 RNP, the mutations accumulated relatively slowly and a broad spectrum of mutations was detected both in planula and polyp stages. In these conditions, functional characterization of gene KO animals would require raising F0 medusae to sexual maturity and crossing to select mutant F1 animals. In contrast, following injection of high doses of Cas9 RNP, only one or two MM deletion were predominant already at the embryonic stage and polyp colonies with very low mosaicism were generated for all 4 genes tested (Fig. 7). The phenotypic consequences of gene knockout could thus already be examined in the F0 generation. A similar bias towards MM deletions has been reported in mammalian cultured cells, the *C. elegans* germ line and in zebrafish^21,30,44,47,48^. Our analysis of published sequence data from CRISPR/Cas9 or TALEN-mediated gene knockout in various metazoan embryos also revealed frequent MM deletion (Fig. S2). Such MM deletions are most likely caused when the DSBs are repaired by MMEJ, a DSB pathway that is less well known than the classic non-homologous end-joining (NHEJ) pathway and the homologous recombination repair pathway (HR).

MMEJ may play a particularly important role during metazoan embryogenesis. Mutant zebrafish in which the key MMEJ pathway component *polQ* is defective become extremely sensitive to double strand breaks caused by Cas9 injection or ionizing radiation^47^. It is thus becoming clear that MMEJ is not a simple backup mechanism for NHEJ. Interestingly NHEJ pathway genes are largely missing in the genome of a pelagic tunicate *Oikopleura dioica*, which exhibits particularly high genome plasticity^49^. The role NHEJ generally plays in metazoan may be at least partly substituted by MMEJ in this species, contributing to genomic instability. Little is however known about the biological significance of MMEJ. *Clytia* can be an interesting research model to study the role of MMEJ during embryogenesis and in polyp growth, in particular during stem cell maintenance. Understanding MMEJ pathway is also important for future development of insertional gene editing^50–52^, which has not been successful so far in *Clytia*. The pattern of induced MM deletion seems different from other species. In *Clytia*, short (2~10 bp) MM deletion are frequently observed. A systematic survey for the induced mutation pattern will facilitate predictions of the dominant genotype, which may be different from predictions proposed in vertebrates^30^. Deep sequencing of mixed target amplicons for a large number of different sgRNAs will provide better understanding of the preferred MM deletions in *Clytia* embryonic cells^44^.

The key but as yet unexplained parameter allowing quasi non-mosaic F0 mutant generation in *Clytia* was the high Cas9 RNP condition. MM deletion occurred in a Cas9 RNP concentration dependent manner during embryogenesis. Our initial hypothesis was that MMEJ pathway activity could be high in the very early stages of the *Clytia* embryogenesis such as cleavage or blastula stages. A high dose of Cas9 RNP would increase the mutagenesis rate and thus more DSBs would be repaired at earlier stages by MMEJ. We however detected no stage specific preference of MM deletion with our dataset. MM deletion was more strongly favoured using high rather than low Cas9 RNP concentrations during early embryogenesis up to 24 hours after injection (Fig. S1E). It is thus more likely that Cas9 concentration affects DNA repair choice rather through cell-intrinsic factors such as the timing of DSB along the cell cycle. For example NHEJ and MMEJ are favoured in G1 phase while homologous recombination (HR) is favoured in S/G2 phase when repair templates are available^53–58^.

MM deletion enrichment in the F0 polyps or jellyfish contrasted with the mosaicism detected at planula stage. The degree of mosaicism greatly reduced between planula and polyp stages (see Fig. 6) even using the low concentration Cas9 RNP. This suggests a stochastic mechanism may be at least partly involved in the reduction of mosaicism. For example, a relatively small number of founder cells may support the polyp colony growth and any mutation generated randomly in these cells will become dominant. This mechanism however does not explain why MM deletions disappear from the polyp colonies using the low concentration Cas9 RNP protocol. Alternatively, or in addition to this, it is possible that MMEJ repair is strongly favoured in the stem cells using the high concentration Cas9 RNP protocol (Fig. 7). It is also possible that stem cells are lost if frequent DSB is repaired by the NHEJ pathway. Currently we lack a reliable method to identify stem cells from *Clytia* and examine the mutations that they carry. Multipotent or pluripotent stem cells called interstitial cells (i-cells) have been identified in other hydrozoans such as Hydra and *Hydratinia echinata*^59,60^ which are required for their regeneration capacity and vegetative growth. In Hydra, i-cells will give rise to neurons, nematocysts, gland cells and germline cells, while epidermal and gastrodermal epithelial cells self-renew independently. I-cells have also been identified in *Clytia* embryos and jellyfish based on the gene expression profile^61^. The DNA repair mechanisms and their regulation in *Clytia* stem cells need to be addressed, as well as further characterisation of stem cell populations in *Clytia* during embryogenesis and in the polyp colony.

There are several intriguing biological questions that could be addressed using *Clytia* as a model animal. We are now able to test the molecular basis of jellyfish behaviour, physiology, ecology and impact on environment. A part of the method established here has been already employed to reveal the role of *Opsin9* gene for light-induced oocyte maturation control^46^. Physiological roles of GFP genes are to be further tested. GFP was identified in a hydrozoan jellyfish *Aqueora victoria* as a bioluminescence protein and several biological roles have been proposed for this protein^36,62,63^. It receives energy from Ca^2+^-activated photo protein Aequorin (Clytin) protein by BRET (bioluminescence resonance energy transfer) then emits bioluminescence, which may be used as a glowing lure for attracting prey in the dim deep sea environment. The presence of GFP in eggs and non-predatory planula suggested the role of green bioluminescence as a camouflage tool by counter illuminating at sea surface. Alternatively the capacity of UV absorbance of GFP itself may be used as UV protection^64^. These hypotheses can now be tested with our *CheGFP1*/*CheGFP2* mutants in a condition modelling their natural habitat. Inactivation of *CheGFP3* and *CheGFP4* genes may also be necessary to address the biological function of *GFP* genes in the jellyfish stage.

In addition, *Clytia* may be a useful model for cell biology and development studies and provide an alternative to classical genetics models. An example is the *CheRfx123* mutant that revealed the role of Rfx protein in sperm flagella. In vertebrates *Rfx1*~*Rfx4* are expressed in developing spermatocytes and spermatids^65^. Mouse mutants of the *Rfx* genes show global defects in spermatogenesis but the mechanisms were not clear^66^. The *CheRfx123* mutant showed for the first time the involvement of Rfx in sperm flagella formation and suggest an evolutionary conserved role for Rfx in cilia/flagella formation in metazoans. *Clytia* can be a useful model for studying genes that play a critical role in in early embryogenesis or reproduction and which may be otherwise difficult to investigate in other species because of mutant lethality. Our approach does not require crossing founder animals to make homozygous mutants, exploiting the prevalence of MM deletion and vegetatively growing mutant polyp colonies as demonstrated in this work.

## Materials and Methods

### Animal culture

Wild type laboratory strains of *Clytia hemisphaerica* Z4B (female) and Z10 (male) were used in this work^33^. All stages are maintained at 19~21 °C in artificial sea water (RedSea salt) dissolved to 37‰ with appropriate water circulation for the jellyfish stage. *Artemia salina* nauplii larvae (1~4 days after hatching) were used for daily feeding.

### Microinjection of Cas9/sgRNA

Cas9 protein was synthesized as described previously^67^. The template vector small guide RNA (sgRNA) was constructed by cloning oligonucleotides into pDR274^22^. sgRNA was synthesized using Mega Shortscript T7 kit (ThermoFisher Scientifique) then purified with ProbeQuant G-50 column (GE healthcare) and ethanol precipitation. The sgRNA was dissolved into distilled water at 80 µM. sgRNA sequences are indicated in the Fig. 3. Purified Cas9 protein was diluted in Cas9 buffer (10 mM Hepes, 300 mM KCl). sgRNA was added to Cas9 protein in excess, up to 1:2 of Cas9:sgRNA prior the injection and incubated for 10 minutes at room temperature. The final concentration was adjusted to 1.0 to 8.0 µM of Cas9 protein. No visible toxicity was observed in embryos injected with up to 30 µM Cas9/sgRNA. The solution was centrifuged at 14,000 rpm for 10 minutes at room temperature, and approximately 3% egg volume injected into eggs before fertilization (Fig. 1).

### Inducing metamorphosis and establishing polyp colonies

Embryos were cultured to the planula larva stage in Millipore filtered sea water (MFSW) at 18 °C. Metamorphosis was induced at 3 or 4 days after injection by transferring 20~100 larvae in 4~5 ml MFSW containing 1 µg/µl synthetic peptide (GNPPGLW-amide, Genscript) spread on a double-width glass slide (75 × 50 mm) and incubating for up to 1 days. Metamorphosing primary polyps fixed on the glass were then transferred to aquariums maintained at 20 °C (favouring male colony development) or 24 °C (favouring female)^68^.

### Microscopic analysis

GFP fluorescence was observed with Olympus BX-51 microscope and Leica DFC310-FX CCD imager, with a fixed exposure condition for each series of imaging. Sperm motility was filmed at 200 frames per second. Flagella curvatures and swimming velocity were measured using image analysis software BohBoh (Bohboh Soft, Tokyo, Japan).

### Estimating knockout efficiency and genotyping

Genomic DNA was extracted using DNeasy blood/tissue extraction kit (Qiagen) from at least 50~500 3-day old planulae from a single injection for each condition or the entire colonies containing at least 20 polyps from each F0 colony grown on a 50 mm × 75 mm slide. Target sequences were amplified by PCR using Phusion DNA polymerase. The list of PCR primers used is in supplementary text. KO efficiency was first estimated to select effective sgRNAs for each target gene by restriction enzyme assay or by TIDE analysis^45^. Alternatively, the PCR products cloned into pGEM-Teasy vector were sequenced. To estimate mutation efficiency for the *CheRfx123* gene (sgRNA: Rfxn1) we used a restriction enzyme AatII (NEB), which cuts only wild type sequence. Similarly, BceAI was used to detect the 5 bp MM deletion obtained with sgRNA Rfxn1 target sequence.

## Electronic supplementary material

Supplementary information
