# Supplementary material for: High doses of CRISPR/Cas9 ribonucleoprotein efficiently induce gene knockout with low mosaicism in the hydrozoan Clytia hemisphaerica through microhomology-mediated deletion
Source: Sci Rep. 2018 Aug 6;8:11734. doi: 10.1038/s41598-018-30188-0 (PMC6078951; doi:10.1038/s41598-018-30188-0)
Supplement: Supplementary file 1 — Supplementary information [file 41598_2018_30188_MOESM1_ESM.pdf]

Supplement Figure 1

High doses of CRISPR/Cas9 ribonucleoprotein efficiently induce gene knockout with low mosaicism in the hydrozoan Clytia hemisphaerica through microhomology-mediated deletion

Tsuyoshi Momose, Anne De Cian, Kogiku Shiba, Kazuo Inaba, Carine Giovannangeli, Jean-Paul Concordet,

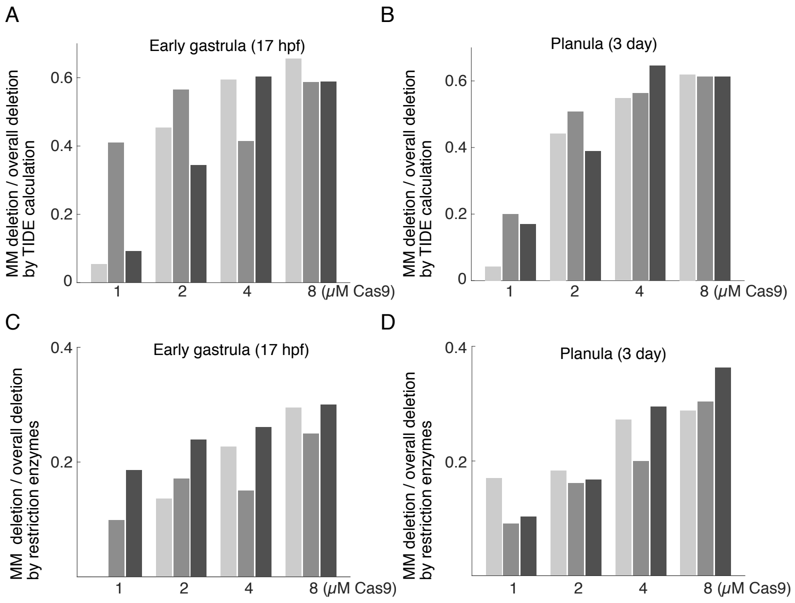

Figure S1

Frequency of the MM deletion frequency among all mutation caused by different Cas9 concentrations. (A, B) MM frequency measured by BceAI and AatII restriction enzymes at 17 hpf early gastrula (A) and 3-day planula (B) stages. (C,D), MM deletion frequency estimated by TIDE analysis in 17 hpf early gastrula (C) and 3-day planula (D) stages.

Supplement Figure 2

High doses of CRISPR/Cas9 ribonucleoprotein efficiently induce gene knockout with low mosaicism in the hydrozoan *Clytia hemisphaerica* through microhomology-mediated deletion

Tsuyoshi Momose, Anne De Cian, Kogiku Shiba, Kazuo Inaba, Carine Giovannangeli, Jean-Paul Concordet,

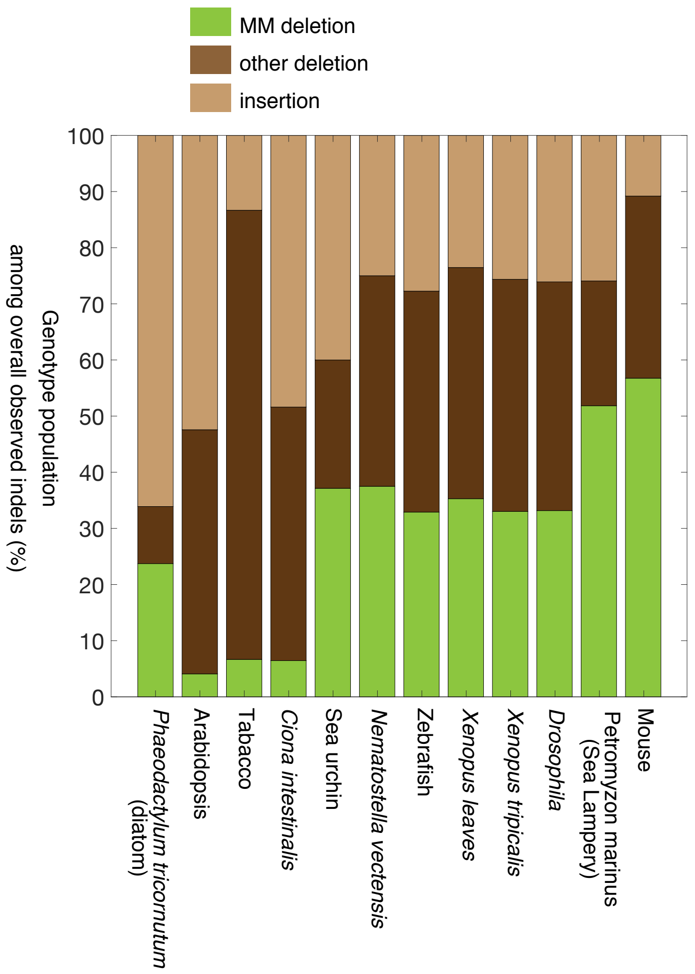

Figure S2 Frequency of Inter-microhomology deletions induced by CRISPR/Cas9 mediated gene KO in different organisms.

Sequence data for this analysis was taken from the following published information, either in the main figure or in the supplementary data. Deletion mutation was classified

Phaeodactylum tricornutum (diatom) doi:10.1038/srep24951
Arabidopsis doi:10.1038/cr.2013.114
Tabacco doi: 10.1093/nar/gkt780
Ciona intestinalis doi:10.1111/dgd.12149 and doi:10.1242/dev.099572
Sea urchin doi:10.1016/j.ydbio.2015.11.018
Nematostella vectensis doi:10.1038/ncomms6486
Zebrafish doi:10.1038/nbt.2501
Xenopos laevis doi:10.1002/dvg.22719
Xenopus tropicalis doi:10.1242/dev.099853
Drosophila doi:10.1016/j.celrep.2013.06.020
Petromyzon marinus (Sea Lampery) doi:10.1242/dev.125609
Mouse doi:10.1038/nbt.1940
